# The Proteolytic Activity of Separase in BCR-ABL-Positive Cells Is Increased by Imatinib

**DOI:** 10.1371/journal.pone.0042863

**Published:** 2012-08-03

**Authors:** Wiltrud Haaß, Michael Stehle, Stefanie Nittka, Michelle Giehl, Petra Schrotz-King, Alice Fabarius, Wolf-Karsten Hofmann, Wolfgang Seifarth

**Affiliations:** 1 Department of Hematology and Oncology, Mannheim Medical Center, University of Heidelberg, Mannheim, Germany; 2 Department of Clinical Chemistry, Mannheim Medical Center, University of Heidelberg, Mannheim, Germany; 3 National Center for Tumor Diseases (NCT), German Cancer Center (DKFZ), Heidelberg, Germany; Institut national de la santé et de la recherche médicale (INSERM), France

## Abstract

Separase, an endopeptidase required for the separation of sister-chromatides in mitotic anaphase, triggers centriole disengagement during centrosome duplication. In cancer, separase is frequently overexpressed, pointing to a functional role as an aneuploidy promoter associated with centrosomal amplification and genomic instability. Recently, we have shown that centrosomal amplification and subsequent chromosomal aberrations are a hallmark of chronic myeloid leukemia (CML), increasing from chronic phase (CP) toward blast crisis (BC). Moreover, a functional linkage of p210BCR-ABL tyrosine kinase activity with centrosomal amplification and clonal evolution has been established in long-term cell culture experiments. Unexpectedly, therapeutic doses of imatinib (IM) did not counteract; instead induced similar centrosomal alterations *in vitro*. We investigated the influence of IM and p210BCR-ABL on Separase as a potential driver of centrosomal amplification in CML. Short-term cell cultures of p210BCR-ABL-negative (NHDF, UROtsa, HL-60, U937), positive (K562, LAMA-84) and inducible (U937p210BCR-ABL/c6 (Tet-ON)) human cell lines were treated with therapeutic doses of IM and analyzed by qRT-PCR, Western blot analysis and quantitative Separase activity assays. Decreased Separase protein levels were observed in all cells treated with IM in a dose dependent manner. Accordingly, in all p210BCR-ABL-negative cell lines, decreased proteolytic activity of Separase was found. In contrast, p210BCR-ABL-positive cells showed increased Separase proteolytic activity. This activation of Separase was consistent with changes in the expression levels of Separase regulators (Separase phosphorylation at serine residue 1126, Securin, CyclinB1 and PP2A). Our data suggest that regulation of Separase in IM-treated BCR-ABL-positive cells occurs on both the protein expression and the proteolytic activity levels. Activation of Separase proteolytic activity exclusively in p210BCR-ABL-positive cells during IM treatment may act as a driving force for centrosomal amplification, contributing to genomic instability, clonal evolution and resistance in CML.

## Introduction

The BCR-ABL tyrosine kinase (TK) formed by the balanced translocation t(9;22)(q34;q11) is the key player in the pathogenesis of chronic myeloid leukemia (CML). Its characteristic deregulated TK activity affects various downstream signaling pathways and results in reprogramming of the prior lineage commitment of hematopoietic stem and early progenitor cells [1]. Compromising multiple aspects of cellular behavior, including proliferation, apoptosis, cell to cell signaling and differentiation, the BCR-ABL oncoprotein triggers aberrant clonal hematopoiesis and drives disease progression from chronic phase (CP) toward the fully transformed phenotype of blast crisis (BC) [2].

Imatinib (IM) is a selective TK inhibitor (TKI) and presents the current first line treatment for CML [3], [4], [5]. Despite significant decreases in BCR-ABL mRNA levels in the bone marrow compartment under IM long-term therapy, persistance of residual CML clones with low BCR-ABL expression and insensitivity to IM treatment has been observed [3]. About 35% of patients in CP develop resistance or intolerance to IM and frequently undergo clonal evolution [6], [7]. Clonal evolution denotes a heterogenous entity of clonal molecular changes in BCR-ABL-positive hematopoietic stem/progenitor cells and has been described in about 30% and 80% of patients in accelerated phase (AP) and BC, respectively [8]. Emergence of altered chromosome numbers, collectively termed aneuploidy, involve an additional derivative chromosome 22, chromosome 17 abnormalities, trisomy 8, and are associated with poor prognosis [9], [10].

Centrosome amplification, in particular, the accumulation of additional centrosomes, is frequently detected in solid and hematological human cancers [11]. It has already been found in pre-neoplastic lesions i.e. early stages of carcinogenesis [11], [12]. Centrosome amplification is the major cause of multipolar mitotic spindle formation and chromosomal missegregation leading to chromosomal instability and aneuploidy [13], [14], [15], [16].

Recently, we have shown that centrosome amplification is an early event in the transformation process of CML and occurs at the earliest identifiable step in CML development [15]. Moreover, in a long-term *in vitro* study on a CML CP model we have established a functional link of p210BCR-ABL TK activity with centrosome amplification and clonal evolution [17]. This was confirmed and further expanded by observations of Patel and Gordon, who found that p210BCR-ABL and c-ABL are both centrosome associated proteins capable of binding to pericentrin, a protein of the pericentriolar matrix. Treatment of CML cells with IM reduced p210BCR-ABL binding to pericentrin [18]. However, IM treatment did not counteract development of centrosome amplification; but IM induced centrosomal and/or cytogenetic alterations in several *bcr-abl*-negative cell line models and *in vivo* [19], [20], [21], [22].

The maintenance of constant centriole numbers in normal proliferating cells is tightly linked to the cell cycle [23], [24]. Disengagement of mother and daugther centriole is a prerequisite for centriole duplication [23] and is provided by proteolytic cleavage of cohesin, a “glue” protein complex that is also responsible for sister-chromatide cohesion [25], [26]. Separase, a cysteine endopeptidase, conducts cleavage of cohesin [25], [27], [28], [29]. Ectopic activation of Separase proteolytic activity causes premature sister-chromatide separation and centriole disengagement [26]. Overexpression of separase has been reported to induce aneuploidy and tumorigenesis [30]. Separase proteolytic activity is tightly regulated by multiple inhibitory mechanisms combining Securin binding, specific serine residue phosphorylation (pSer1226) by CyclinB1/Cdk1, PP2A binding and autocatalytic cleavage [31], [32], [33], [34], [35]. The finding that separase is overexpressed in several cancers, including CML renders this protease a key subject of investigation to unravel the molecular mechanisms involved in the development of centrosome amplification in IM-treated CML [18], [30], [36], [37].

In this study, we set out to analyze the short-term effects of IM on the “oncogene” separase in BCR-ABL-positive and -negative cells. We employed a panel of human cell lines varying in p210BCR-ABL expression levels that served as models for different stages of CML. We report on separase transcription, protein expression, and Separase proteolytic activity. Furthermore, proteins of the corresponding master regulatory pathways were analyzed. We observed a post-translational activation of Separase proteolytic activity in BCR-ABL-positive cells after treatment with therapeutic IM doses. The potential clinical impact was discussed.

## Results

### Study design and cell line characterization

To analyze the conditional context between p210BCR-ABL, separase activity and IM treatment, we performed cell culture experiments using a panel of six well established human cell lines (Table 1). Primary NHDF cells and SV-40 immortalized UROtsa served as models for human non-malignant cells. While U937 and HL-60 represent *bcr-abl*-negative malignant cells, K562 and LAMA-84 are well established model systems for CML BC. U937p210BCR-ABL/c6 cells with inducible p210BCR-ABL expression (Tet-On) display one single *bcr-abl* transgene with moderate p210BCR-ABL expression in the (Doxycycline-) induced state and served as a model of CML CP [17].

**Table 1:** Origin and characteristics of human cell line models under investigation.

Cell line	Cell type	Origin	Doubling time [h]^1^	BCR-ABL-copy no/type	Cytogenetics	Used as model for
NHDF	normal human dermal fibroblasts, primary	juvenile foreskin, from healthy donor	∼72	none	46,XY	normal cells
UROtsa	human urothelial	normal urothelial cells immortalized with SV40	∼63	none	46,XX	normal cells
HL-60	human AML-M2	PB of a patient with AML-M2	∼40	none	82–88<4n>XX,−X,−X,−8,−8,−16,−17,−17,+18,+22,+2mar, ins(1;8)(p?31;q24hsr)x2,der(5)t(5;17)(q11;q11)x2,add(6) (q27)x2,der(9)del(9)(p13)t(9;14)(q?22;q?22)x2,der(14) t(9;14)(q?22;q?22)x2,der(16)t(16;17)(q22;q22)x1-2, add(18)(q21) - sideline add(18)(q21) - sideline with: −2,−5,−15,del(11)(q23.1q23.2) - c-myc amplicons present in der(1) and in both markers (DSMZ)	*bcr-abl*-negative leukemia
U937p210BCR-ABL/c6	human histiocytic lymphoma with recombinant p210BCR-ABL gene	pleural effusion of a patient with generalized histiocytic lymphoma, stable transfected with p210BCR-ABL cDNA construct under Tet-On promoter control [17]	37 (ON)^2^	1/(b3a2)	47∼94,X,−Y,+1,t(1;6;1),t(1;16),+2,t(2;5),+4,del(4q),+5, del(5p),der(6)t(6;19)del(6p),+7,+7,t(9;17),−10,t(10;13), del(11q),+13,add(13q),t(14;15),+15,+15,+16,+17[cp10]	CML-CP
LAMA-84	human CML (BC)	PB of a patient with CML BC	∼30	4/(b3a2)	73/74(69–77)<3n>XX,−X,+1,−2,+5,+6,del(7)(p15),+8, der(9)t(9;22)(q34;q11)x2,i(11q),+13,add(13)(q33),−14,+17,+17,del(17)(p12),−18,+22, der(22)t(9;22)(q34;q11)x4,+mar, (DSMZ)	CML-BC
K562	human CML (BC)	pleural effusion of a patient with CML in BC	∼34	11/(b3a2)	61–68<3n>XX,−X,−3,+7,−13,−18,+3mar,del(9)(p11/13), der(14)t(14;?)(p11;?),der(17)t(17;?)(p11/13;?),der(?18) t(15;?18)(q21;?q12),del(X)(p22) (DSMZ)	CML-BC

Abbreviations: PB, peripheral blood; CML, chronic myeloid leukemia; CP, chronic phase; BC, blast crisis; no, number; AML-M2, acute myeloid leukemia M2; DSMZ, German Collection of Microorganisms and Cell Cultures, Braunschweig, Germany.

1 doubling time of subconfluent cells when 3×10^5^ cells were seeded in 100 mm cell culture dishes.

2 p210BCR-ABL Tet-ON promoter induction with Doxycycline [1 µg/ml].

As a continuation of our previous studies on long-term cell cultures [17], where we found that prolonged treatment with IM induced centrosomal and cytogenetic alterations in several *bcr-abl*-negative cell lines, we performed short-term cell culture experiments to assess the impact of therapeutic doses of IM on expression and proteolytic activity of Separase. Focusing on changes occurring within the first few rounds of the cell cycle after IM administration, our experimental setting should provide insight into the post-translational regulatory mechanisms elapsing before any phenotypic alterations in centrosomal or cytogenetic status may become detectable. Since the proteolytic activity of Separase is regulated in a tight cell cycle-dependent manner, treatment periods were chosen with respect to the respective cell doubling times so that less than two cell cycle rounds were completed under IM treatment (Table 1) and less than 15% of cells were apoptotic. Accordingly, we assigned 6 d (NHDF, UROtsa), 48 h (HL-60, U937, U937p210BCR-ABL/c6-On) and 24 h (K562, Lama-84) of treatment as appropriate before cell harvesting and target analysis (Table 1).

All cell lines were treated with therapeutic doses of IM (range: 0.5 to 10 µM) as performed in our previous studies [17], [19], [20]. In accordance with data from extensive studies on the dose-dependent effects and time kinetics of IM [38], [39], [40] we applied lower IM doses (range: 0.5 µM to 2.5 µM) for leukemia-derived BCR-ABL-positive cells (K562 and LAMA-84) than for BCR-ABL-negative cells (range: 2.5 µM to 10 µM). K562 and LAMA-84 reacted highly sensitively to IM in terms of proliferation and survival rates. Treatment with IM doses higher than 2.5 µM for a longer period than 24 h impeded the collection of enough viable cells for Western Blot analysis, qRT-PCR and Separase activity assays (data not shown).

**Figure 1:**
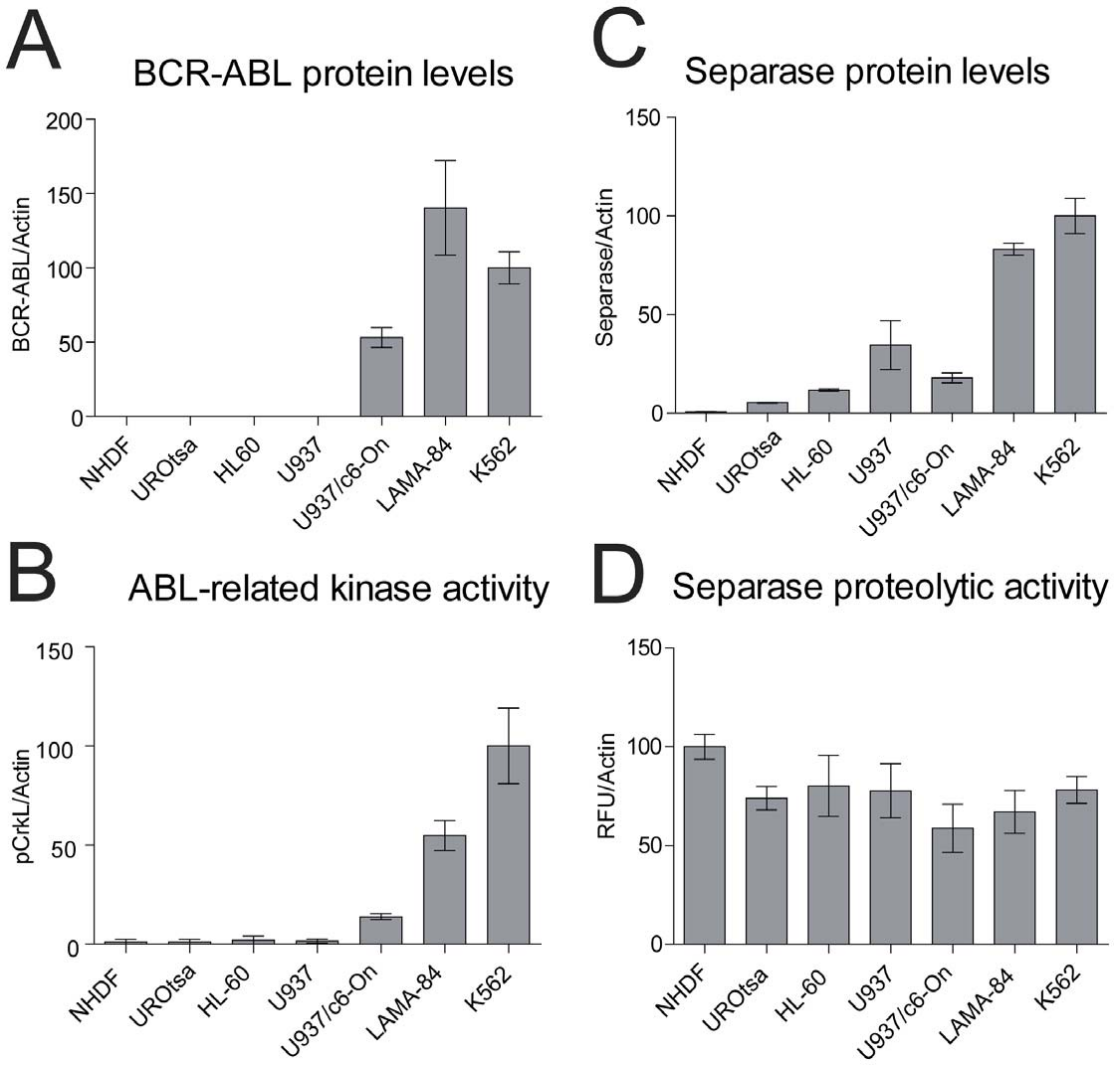
Protein and activity levels of BCR-ABL and Separase in cell lines under investigation. Protein levels of p210BCR-ABL (**A**) and Separase (**C**) based on densitometric evaluation of immunostained Western blots were normalized to Actin as loading control. Abl-related TK activity (BCR-ABL+c-ABL) was measured as pCrkL/Actin (**B**). Separase proteolytic activity (**D**) was quantified using an *in vitro* fluorometric assay and was given as relative fluorescence units/Actin (RFU/Actin). Analyses were performed on protein lysates derived from p210BCR-ABL-positive (LAMA-84, K562) and -negative cells (NHDF, UROtsa, HL-60, U937), and from induced U937p210BCR-ABL/c6 cells (U937/c6-On) expressing a p210BCR-ABL transgene under control of a Doxycycline inducible promoter.

Initially, all untreated cell lines were tested thoroughly with respect to their identity (all confirmed), karyotype and centrosome status, and proliferation rate (Table 1). Protein levels and proteolytic activity levels of Separase and p210BCR-ABL TK activity (pCrkL) were evaluated (Figure 1). As expected, p210BCR-ABL protein was detected exclusively in *bcr-abl*-positive cell lines (Figure 1A). LAMA-84 and K562 displayed high levels of p210BCR-ABL protein (140+/−31.8% and 100.0+/−10.8%, respectively) followed by U937p210BCR-ABL/c6-On cells (53.1+/−6.6%) after induction with Doxycycline for 48 h [17]. Densitometric analysis of pCrkL as a surrogate marker for p210BCR-ABL TK activity (Figure 1B) revealed the highest phosphorylation levels in K562 ( = 100% phosphorylation), followed by LAMA-84 (54.9+/−7.5%) and U937p210BCR-ABL/c6-On cells (13.8+/−1.5%), the latter showing a 9-fold increase of pCrkL phosphorylation with respect to the parental cell line U937 lacking p210BCR-ABL transgene expression. Minor phosphorylation levels for pCrkL were detected in NHDF (3.6+/−1.2%), UROtsa (2.5+/−1.2%), HL-60 cells (4.1+/−2.1%) and U937 cells (1.5+/−1.0).

Separase protein level analysis revealed a general overexpression (range 27- to 151-fold) in all BCR-ABL-positive cells when compared to NHDF cells (Figure 1C). This is in line with various reports on separase overexpression in cancers, including CML [18], [36]. Moreover, Separase protein levels correspond to observed doubling times and p210BCR-ABL TK activity, as fast-growing cells (K562 or LAMA-84) display higher Separase protein levels (relative protein levels of 100.0+/−9.0% and 83.1+/−3.1%, respectively) than slow-growing cells (NHDF 72 h doubling time, relative protein level 0.66+/−0.15%; UROtsa 63 h doubling time, relative protein level 5.3+/−0.2%). Notably, in spite of differences in doubling times (Table 1) and Separase protein levels (Figure 1C) all exponentially growing cells display comparable levels of Separase proteolytic activity (Figure 1D). This suggests that separase expression correlates positively with p210BCR-ABL TK activity, whereas regulation of Separase proteolytic activity is independent of p210BCR-ABL.

### Separase protein levels and Separase proteolytic activity are decreased in BCR-ABL-negative cells under IM treatment

**Figure 2:**
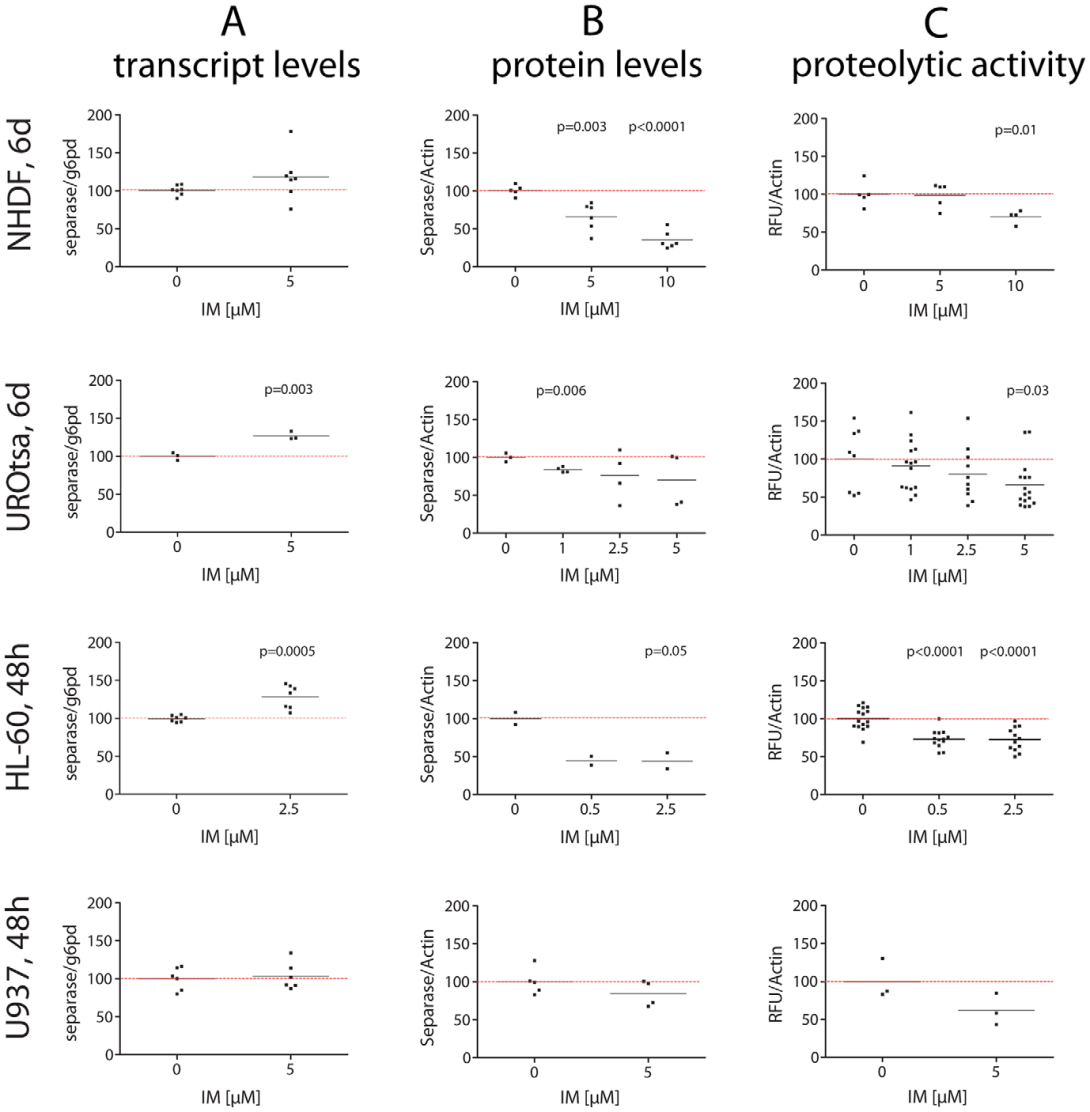
Transcript levels, protein levels and proteolytic activity of Separase in BCR-ABL-negative cells treated with IM. Cells were treated individually with distinct concentrations (0.5 to 10 µM) of IM. After about two cell cycle rounds (NHDF, UROtsa, 6 d; HL-60 and U937, 48 h) total RNA and protein lysates were prepared and analyzed by qRT-PCR (A), Western blot immunostaining (B) and Separase fluorometric activity assays (C). For Westren blot experiments, Actin served as loading control and/or for densitometric data normalization. Each data point corresponds to one single experiment. Only significant p-values as calculated between treated and untreated cells were shown (see Table 2 for summarized Δ-values). For a representative set of corresponding immunostained Western blots compare Figure 5 panel B.

For all BCR-ABL-negative cells (NHDF, UROtsa, HL-60, U937) a dose-dependent decrease in Separase protein levels was observed after IM exposure (Figure 2B, Table 2). Protein levels dropped (range 15 to 34%) at IM concentrations between 2.5 to 5 µM. HL-60 cells resulted more sensitive showing a decrease of 55.8+/−13.3% at a concentration of 2.5 µM IM.

**Table 2:** Percent changes (Δ-values) in transcript levels, protein levels and proteolytic activity of Separase after IM treatment when compared to the corresponding untreated cells.^1^^,^ 2

Cell line, period of treatment	Transcript levels [separase/g6pd]	Protein levels [Separase/Actin]	Proteolytic activity [RFU/Actin]
**NHDF, 6 d**	+17.3±12.0 (5 µM, p = 0.2)	−34.4±8.6 (5 µM, p = 0.003)	−1.3±10.1 (5 µM, p = 0.9)
**UROtsa, 6 d**	+26.84±4.2 (5 µM, p = 0.003)	−30.0±21.0 (5 µM, p = 0.2)	−34.2±15.0 (5 µM, p = 0.03)
**HL60, 48 h**	+28.6±6.1 (2.5 µM, p = 0.0005)	−55.8±13.3 (2.5 µM, p = 0.05)	−27.6±5.8 (2.5 µM, p<0.0001)
**U937, 48 h**	+3.5±9.5 (5 µM, p = 0.7)	−15.3±11.5 (5 µM, p = 0.2)	−38.2±19.3 (5 µM, p = 0.1)
**K562, 24 h**	−36.4±13.6 (1 µM, p = 0.06)	−56.9±7.4 (1 µM, p = 0.002)	+9.1±3.2 (1 µM, p = 0.01)
**LAMA-84, 24 h**	−90.3±6.1 (2.5 µM, p<0.0001)	−75.8±16.8 (2.5 µM, p = 0.006)	+31.1±14.7 (2.5 µM, p = 0.05)
**U937/c6-On, 48 h**	−41.2±12.0 (5 µM, p = 0.03)	−56.8±12.3 (5 µM, p = 0.0002)	+26.1±8.9 (5 µM, p = 0.03)

1 Δ-values were calculated from significant data sets diagramed in Figures 2 and 4.

2 for representative corresponding immunostained Western blots see Figure 5 panels B and C.

Abbreviations: RFU, relative fluorescence units; d, days; h, hours; IM, imatinib; +, increase; −, decrease; U937/c6-On, U937p210BCR-ABL/c6 cells with switched on transgene promoter.

Separase proteolytic activity seems tightly linked to protein levels as dose-dependent decreases in proteolytic activity were found in all IM-treated cell lines (Figure 2C). Relative Separase activity losses of 1.3+/−10.1%, 34.2+/−15.0%, 27.6+/−5.8% and 38.2+/−19.3% were observed in NHDF, UROtsa, HL-60 and U937 cells at concentrations of 5, 5, 2.5, and 5 µM IM, respectively (Table 2).

**Figure 3:**
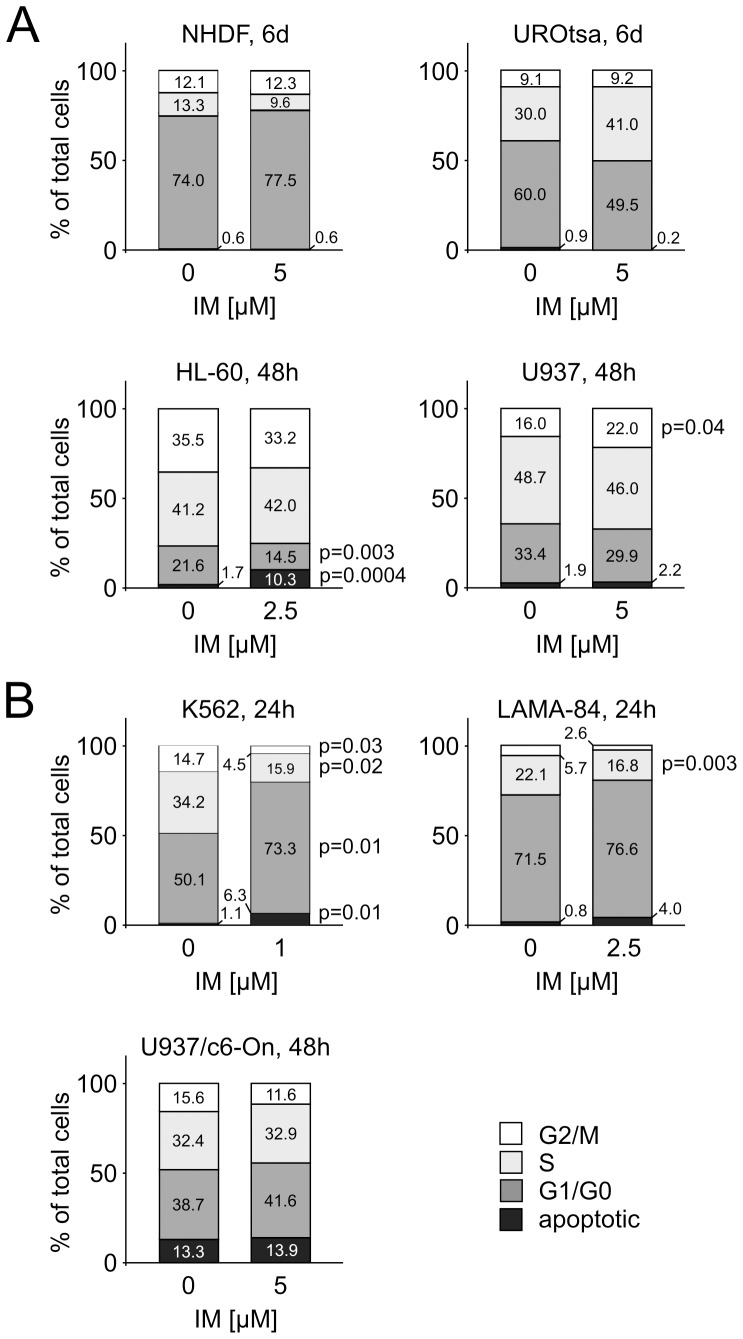
Cell cycle profiles of untreated and IM-treated cell lines. Triplicate cell cultures were incubated with IM at times and doses where significant changes in Separase protein levels and/or Separase proteolytic activation have been observed (Figure 2 and 4). Subsequently, the cell cycle profile was analyzed by flow cytometry after propidium iodide staining. The percentages of cells in G2/M, S, G1/G0 phases and in apoptotic state are depicted in each bar.

One might assume that the observed effect could be due to IM-related delay in the cell cycle, i.e. decreased proportion of cells entering anaphase, where separase activation occurs. However, FACS analysis of NHDF, UROtsa, HL-60 and U937 cells revealed no significant decreases of G2/M cell proportion under IM treatment (Figure 3A). Rather, IM determined an approximate 6% increase of the G2/M fraction of total U937 cells (p = 0.04).

The corresponding separase transcript levels as analyzed by qRT-PCR (Figure 2A, Table 2) were stable (NHDF, U937) or showed increases after IM exposure (UROtsa, HL-60). This observation suggests that the observed IM-associated differences in Separase protein levels are not due to transcriptional regulation. Regulation may be a matter of translation and/or protein stability.

### Separase protein levels are decreased, but Separase proteolytic activity is increased in BCR-ABL-positive cells under IM treatment

**Figure 4:**
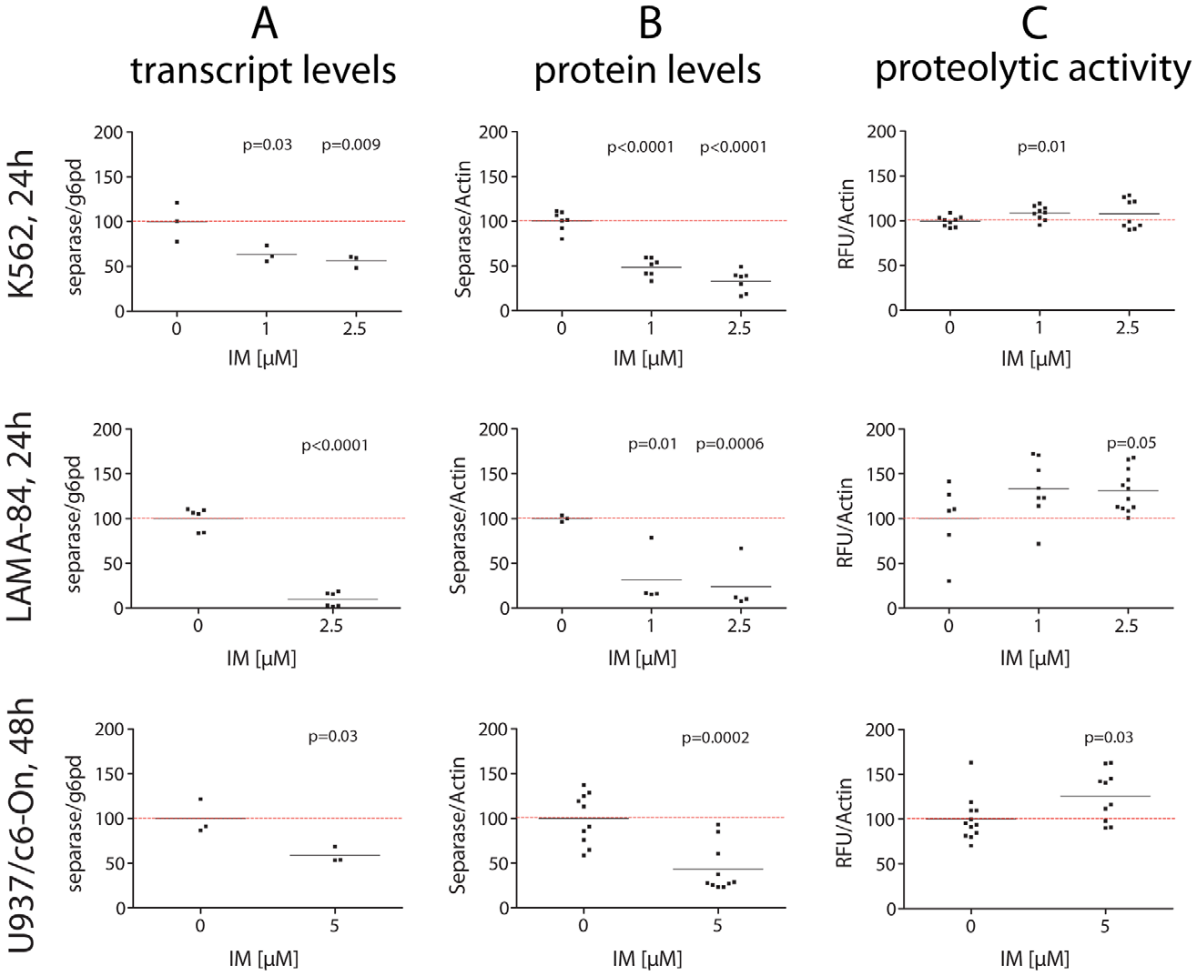
Transcript levels, protein levels and proteolytic activity of Separase in BCR-ABL-positive cells treated with IM. Cells were treated individually with distinct concentrations (0.5 to 5 µM) of IM. After about two cell cycle rounds (K562, LAMA-84, 24 h; U937p210BCR-ABL/c6-On, 48 h) total RNA and protein lysates were prepared and analyzed by qRT-PCR (A), Western blot immunostaining (B) and separase fluorometric activity assays (C). For Western blot experiments, Actin served as loading control and/or for densitometric data normalization. Each data point corresponds to one single experiment. Only significant p-values as calculated between treated and untreated cells were shown (see Table 2 for summarized Δ-values). For a representative set of corresponding immunostained Western blots compare Figure 5 panel C. Abbreviations: U937/c6-On, U937 cells expressing a p210BCR-ABL transgene (Tet-On system) after induction with Doxycycline (U937p210BCR-ABL/c6-On).

Analogous experiments were performed with the BCR-ABL-positive cell lines (Figure 4). Compared to BCR-ABL-negative cell lines, the genuine CML BC-derived cell lines K562 and LAMA-84 displayed conspicuous sensitivity to IM after 24 h. Considerable decreases in Separase protein levels were achieved for K562 and LAMA-84 with low doses (1 µM) of IM (Figure 4B) pointing to the strong proliferative BCR-ABL-dependency of these cell lines as discussed by others [38]. In contrast, p210BCR-ABL-expressing U937p210BCR-ABL/c6-On cells are less sensitive, showing 56.8+/−12.3% (5 µM IM after 48 h) decrease in the Separase protein levels (Figure 4B). In contrast to all BCR-ABL-negative cell lines, transcript levels of K562, LAMA-84 and U937p210BCR-ABL/c6-On cells under IM treatment decreased (Figure 4A). This is best explained by the direct effect of IM on cell proliferation and cell survival. Cell cycle analysis revealed enlargement of apoptotic cell proportion and decreases in G2/M cell counts (Figure 3B).

Unexpectedly, despite the observed decrease in separase transcript and Separase protein levels, increased levels of Separase proteolytic activity were measured (Figure 4C). Increases of 9.1+/−3.2% and 31.1+/−14.7% were observed in K562 and LAMA-84 cells at IM doses of 1 and 2.5 µM, respectively (Table 2). An increase was also observed in U937p210BCR-ABL/c6-On cells upon 5 µM IM administration (26.1+/−8.9%).

As a result, about 25% of the residual Separase protein perform about 130% proteolytic activity in LAMA-84 cells (as calculated from Table 2) meaning an approximate 5-fold increase in Separase activity when compared to the respective untreated cells. Thus, the inhibitory effect of IM on Separase protein expression seems to be counterbalanced by the increase in Separase proteolytic activity. In fact, this compensation leads to a 31% increase in overall Separase proteolytic activity (Table 2). No changes have been detected in intracellular localization of Separase and in the centrosomal status throughout the respective observation periods (data not shown).

### The increase of Separase proteolytic activity in BCR-ABL-positive cells concurs with changes in respective regulatory pathways

**Figure 5:**
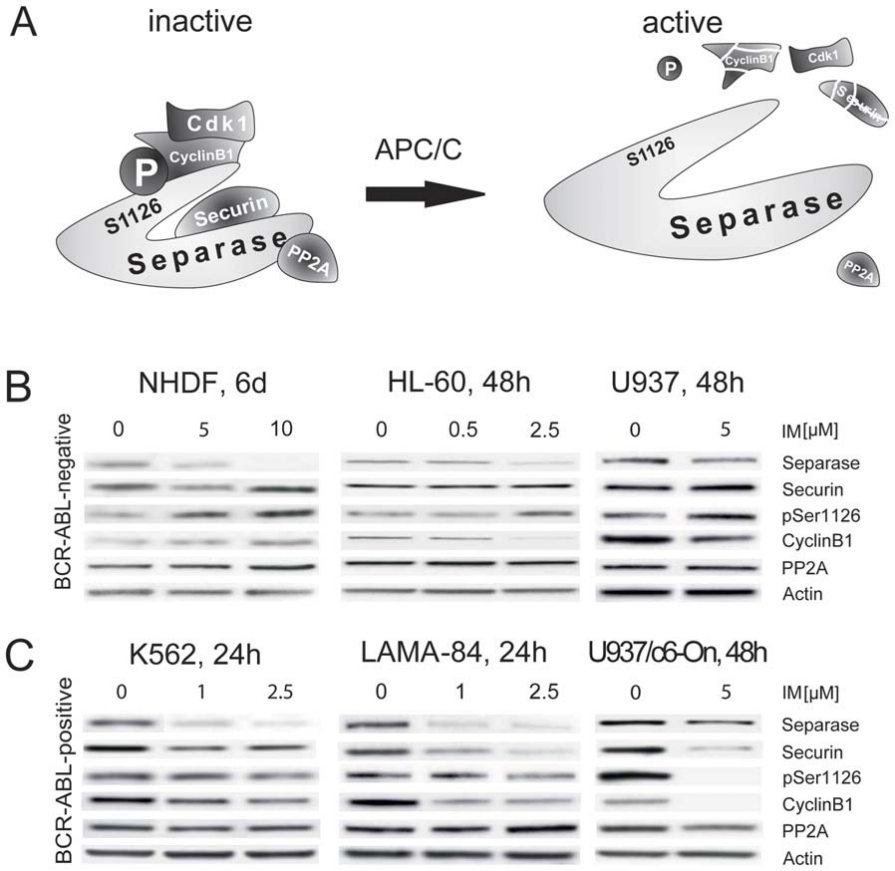
Analysis of master Separase proteolytic activity regulators in BCR-ABL-negative and -positive cell lines treated with IM. Schematic diagram of cooperating inhibitory factors that regulate Separase proteolytic activity in a tight cell cycle controlled manner (**A**). Degradation of Securin, inactivation of the CyclinB1/CDK1 complex, dephosphorylation of Separase at a specific serine residue (pSer1126) by the anaphase promoting complex/cyclosome (APC/C), and the release of PP2A contribute to activation of Separase. Representative composite image of Western blot immunostaining experiments illustrate the expression levels of Separase and relevant regulatory proteins (Securin, pSer1126, CyclinB1 and PP2A) in BCR-ABL-negative (**B**) and BCR-ABL-positive (**C**) cell lines. Images are cropped sections derived from stripped and reprobed Western blot immunostainings used for acquisition of densitometric data shown in Figures 2 and 4. Cells were treated with IM for times and doses given on top. Actin served as loading control. The densitometric data of at least triplicate experiments are presented in Table 3.

To address the potential molecular mechanisms of how IM enhances the proteolytic activity of Separase in BCR-ABL-positive cells, we analyzed the expression levels of respective relevant regulatory proteins. Securin and PP2A both bind to Separase and thereby inhibit proteolytic activity (Figure 5A). CyclinB1/Cdk1-dependent kinase phosphorylation of Separase at amino acid residue serine 1126 ( = pSer1126) constitutes an essential inhibiting mechanism of Separase activity and was assessed by means of pSer1126-specific antibody staining.

Comparison of BCR-ABL-negative cells (Figure 5B) with BCR-ABL-positive cells (panel C) revealed stable or increased inhibitor levels (Securin, pSer1126, CyclinB1, PP2A) in the former, and drug-related decreases in most of the latter. For example, LAMA-84, when compared to HL-60, displayed striking decreases in Securin (−82.1+/−5.1%), pSer1126 (−20.9+/−4.6%) and CyclinB1 (−34.2+/−15.3%) protein levels (Table 3). These data suggest that IM treatment triggers degradation of Securin in BCR-ABL-positive cells. Activation of this main regulatory pathway, including loss of the specific phosphorylation at serine residue 1126 (pSer1126) by parallel degradation of CyclinB1, is associated with activation of Separase.

**Table 3:** Percent changes (Δ-values) in regulator levels of Separase proteolytic activity after IM treatment when compared to the corresponding untreated cells.^1^

Cell line, period of treatment	Securin protein levels^2^	Separase phosphorylation (pSer1126)^2^^, ^ 3	CyclinB1 protein levels^2^	PP2A protein levels^2^
**NHDF, 6 d**	+67.7±40.2 (5 µM, p = 0.1)	+77.9±29.7 (5 µM, p = 0.03)	+59.4±46.6 (5 µM, p = 0.3)	+27.3±22.4 (5 µM, p = 0.3)
**UROtsa, 6 d**	+27.3±21.8 (5 µM, p = 0.3)	−9.1±16.0 (5 µM, p = 0.6)	+36.4±49.6 (5 µM, p = 0.5)	−11.9±52.5 (5 µM, p = 0.8)
**HL60, 48 h**	+3.5±6.7 (2.5 µM, p = 0.6)	+53.0±15.3 (2.5 µM, p = 0.04)	−15.7±35.3 (2.5 µM, p = 0.7)	+37.3±51.5 (2.5 µM, p = 0.5)
**U937, 48 h**	+21.4±19.5 (5 µM, p = 0.4)	+76.4±44.3 (5 µM, p = 0.1)	−18.5±26.9 (5 µM, p = 0.5)	−2.8±9.8 (5 µM, p = 0.8)
**K562, 24 h**	−62.3±9.1 (1 µM, p = 0.02)	−26.6±34.5 (1 µM, p = 0.5)	−9.7±25.5 (1 µM, p = 0.7)	−19.6±20.8 (1 µM, p = 0.4)
**LAMA-84, 24 h**	−82.1±5.1 (2.5 µM, p<0.0001)	−20.9±4.6 (2.5 µM, p = 0.006)	−34.2±15.3 (2.5 µM, p = 0.07)	−17.2±14.2 (2.5 µM, p = 0.3)
**U937/c6-On, 48 h**	−46.7±2.3 (5 µM, p = 0.003)	−41.4±17.3 (5 µM, p = 0.05)	−49.7±15.6 (5 µM, p = 0.005)	−30.5±8.1 (5 µM, p = 0.06)

1 for representative corresponding immunostained Western blots see Figure 5 panels B and C.

2 after normalization to Actin protein levels.

3 Phosphorylation at serine residue 1126 of Separase.

Abbreviations: RFU, relative fluorescence units; d, days; h, hours; IM, imatinib; +, increase; −, decrease; U937/c6-On, U937p210BCR-ABL/c6 cells with switched on transgene promoter.

## Discussion

Since Separase is one of the master key players in centriole duplication, and overexpression has been associated with formation of supernumerary centrosomes in cancers including CML [18], [23], [30], [36], we investigated the influence of BCR-ABL TK on separase in the therapeutic context of IM. We analyzed Separase on multiple regulatory levels of expression, i.e. transcriptional, translational and post-translational levels, in a panel of six well characterized and widely accepted human cell lines. Of these, K562, LAMA-84 and U937p210BCR-ABL/c6 displayed different levels of p210BCR-ABL protein and, therefore, mimic the different stages of CML (Figure 1) [17], [38], [41], [42]. Since each cell line is unique with respect to karyotype, BCR-ABL copy number, cell cycling (doubling) time and IM sensitivity, each cell line was treated individually according to its unique growth and sensitivity behaviour. A distinct IM dose and time schedule was applied, where lower IM doses and incubation times were applied for fast-growing, BCR-ABL growth-dependent, cells (K562 and LAMA-84) than for BCR-ABL-positive slow-growing cells (U937p210BCR-ABL/c6-On) and BCR-ABL-negative cells (NHDF, UROtsa, HL-60, U937). This treatment schedule allowed for preparation of RNA and protein lysates in sufficient amounts and quality to perform the presented qRT-PCR, Western Blot experiments and Separase activity assays.

We found that regulation of separase in IM-treated BCR-ABL-positive cells is complex and occurs on both protein expression and proteolytic activity levels. i) Treatment of BCR-ABL-negative cells (NHDF, UROtsa, HL-60, U937) with IM strongly pointed to a regulation of Separase protein expression on levels of translation and/or protein stability rather than transcription, as transcript and protein level changes did not coincide upon IM application (Figure 2). This may also be true for BCR-ABL-positive cells, although concomitant transcript and protein level decreases were observed after IM application (Figure 4). We surmise that this coincidence may be due to the antiproliferative and proapoptotic effect of IM in BCR-ABL-positive cells (K562 and LAMA-84) as supported by the observed cell cycle profiles of IM-treated and untreated cell (Figure 3). IM treatment resulted in considerable decreases in the proportion of G2/M and S phase cells, whereas the amount of apoptotic cells increased.

ii) Post-translational regulation on the proteolytic activity level becomes evident when all untreated cell lines under investigation were compared with respect to BCR-ABL TK activity, Separase protein levels and Separase proteolytic activity (Figure 1). While Separase protein expression correlated positively with p210BCR-ABL TK activity as reported by others [18], [36], and was in fact highest in K562 and LAMA-84, all exponentially growing cells displayed about the same proportion of Separase proteolytic activity (Figure 1D). This strongly suggests that regulation of Separase proteolytic activity is independent of p210BCR-ABL whereas Separase protein expression is linked to BCR-ABL TK activity. Our experiments demonstrate that IM application can affect both levels of Separase regulation.

Decreased Separase protein levels were observed in all investigated cell lines after IM application. This effect is BCR-ABL-independent as it was equally observed in both BCR-ABL-positive and negative cells. Except for BCR-ABL-positive cells, decreased Separase proteolytic activity levels were observed in all p210BCR-ABL-negative cell lines. FACS analyses revealed that the parallel changes in Separase protein and activity levels are not associated with changes in the proportion of G2/M cells. Decreased Separase protein level may be related to decreased translation and/or enhanced degradation of Separase protein. Reduced Separase proteolytic activity may be best explained by a reduced proportion of cells entering mitotic anaphase, where the protease is regularly activated by the anaphase-promoting complex/cyclosome (APC/C) [43]. Since our FACS analyses revealed no changes (NHDF, UROtsa, HL-60), or an 6% increase in G2/M cells after IM treatment (U937, Figure 3), we assume that the majority of cells were on hold at the G2/M check point before the transition to M phase [44]. An IM-induced G2/M arrest has been reported previously for various cancer cells [45].

The second level of regulation (proteolytic activity level) was exclusively affected by IM in p210BCR-ABL-positive cells (K562, LAMA-84, U937p210BCR-ABL/c6-On). We observed increased Separase proteolytic activities despite lowered Separase protein levels after IM application. This unexpected activation (LAMA-84, 5.4-fold; K562, 2.5-fold; U937p210BCR-ABL/c6-On, 2.9-fold; calculated as a ratio [Separase protein/Separase proteolytic activity] from data given in Table 2, suggests a BCR-ABL-dependent compensatory mechanism that is able to counterbalance the inhibitory effect of IM on Separase protein expression by raising the proteolytic activity of Separase.

For a mechanistic explanation we conclude that Separase was activated regularly via the APC/C, a large multi-subunit complex operating as a specific E3 ubiquitin ligase [46], [47], because in all cell lines with increased Separase proteolytic activity (K562, U937, U937p210BCR-ABL/c6-On), we measured decreased protein levels of Securin, pSer1126 and CyclinB1 (Figure 5 and Table 3). APC/C promotes the metaphase/anaphase transition by ubiquitizing and degrading Securin, the main inhibitor of Separase proteolytic activity. Moreover, APC/C also ubiquinates CyclinB1 and accelerates its degradation during late mitotic phase, which results in activation of Separase and mitotic exit [48]. Dysregulation of APC/C-dependent proteolysis of these substrates is considered to contribute to mitotic catastrophe and tumorigenesis [43]. The activity of APC/C is regulated by a complex network of antagonistic phosphorylating events of its subunits resulting in CDC20 binding, one of its main activating subunits. We hypothesize that IM targets one or more phosphoproteins of the APC/C, thereby activating the E3 ubiquitin ligase function. This may favor the degradation of Securin and CyclinB1, and selective dephosphorylation of Separase at serine residue 1126 (pSER1126). Finally, this may lead to activation of Separase. The explanation of why Separase activation is exclusively observed in BCR-ABL-positive cells remains elusive. However, a potential mechanistic link is provided by a previous microarray study reporting that BCR-ABL expression promotes overexpression of CDC20 [49] and thereby enables activation of the APC/C [43], [50]. We further suggest that this Separase activating effect, observed exclusively in BCR-ABL-positive cells, is not attributed to BCR-ABL TK activity, but to the protein itself as we consider the applied IM concentrations high enough for almost complete inhibition of ABL-related TK activity in our experiments (IC50 in cellular assays: 280 nM) [39]. Therefore, protein-protein interaction rather than ABL-related TK activity may be responsible for the observed effects. This might be favored by the coiled-coil domain of the BCR protein that remains in the BCR-ABL fusion protein and promotes dimerization of p210BCR-ABL or perhaps binding to other proteins [51].

There is a potential clinical impact of our observation. We hypothesize that the increased proteolytic activity of Separase may be a trigger for unscheduled centriole duplication and subsequent centrosomal amplification that probably contributes to chromosomal missegregation and the development of genomic instability during further cell cycles. This assumption is concordant with the molecular pathology of CML and also with our earlier observations [17], [19]. Clonal evolution with consistent chromosomal aberrations, in addition to the t(9;22)(q34;q11), is frequently detected in 30% of patients with AP and about 80% patients in BC [10]. Development of resistance in patients undergoing IM therapy frequently concurs with clonal evolution, which points to clonal evolution as a mechanism of resistance [10], [52]. Furthermore, under IM, the outcome of patients with clonal evolution is significantly inferior compared to those without [9]; suggesting a close conditional interrelationship to IM treatment. It is therefore tempting to speculate that the IM-related upregulation of Separase proteolytic activity in BCR-ABL-positive cells may play a role as a promoting mechanism for the development of tumor heterogeneity. Even in dormant BCR-ABL- low-expressing clones, such as quiescent stem cells [3], this may eventually create descendant cell populations with enhanced fidelity to escape therapeutic pressure.

In summary, we found that the regulation of Separase in IM-treated BCR-ABL-positive cells occurs on both protein expression and enzyme activity levels. Furthermore, we established a mechanistic link between IM treatment, BCR-ABL expression and increased Separase proteolytic activity. Our in vitro study has provided a hypothesis of how BCR-ABL-positive cells undergoing IM therapy may trigger centrosomal amplification and genomic instability. In CML patients during IM treatment, enhanced Separase proteolytic activity in *bcr-abl*-positive stem and progenitor cells with residual BCR-ABL protein expression may promote tumor heterogeneity, clonal evolution and development of resistance. We believe that future studies on the Separase regulatory network in CML may give rise to new concepts in carcinogenesis and leukemia therapy.

## Materials and Methods

### Cell lines and culture conditions

Six human cell lines (NHDF, UROtsa, HL-60, K562, LAMA-84, U937, U937p210BCR-ABL/c6^Tet-ON^) were analyzed (Table 1). NHDF and U937 were derived from Promocell GmbH (Heidelberg, Germany). HL-60, K562 and LAMA-84 were obtained from the DSMZ (German Collection of Microorganisms and Cell Cultures, Braunschweig, Germany). UROtsa were obtained from the Department of Urology, Mannheim Medical Center, University Heidelberg, Mannheim, Germany and were cultured as described previously [53]. The U937 monocytic cell line clone c6 (U937p210BCR-ABL/c6) expressing p210BCR-ABL under the control of a Tet-On system was propagated as described previously [17]. The p210BCR-ABL expression was induced by addition of 1 µg/ml Doxycycline (Gibco/Invitrogen, Karlsruhe, Germany) to standard medium. Cell line authentication was performed by DNA profiling commissioned at the DSMZ. All other cells were cultured in RPMI-1640 medium (Gibco/Invitrogen), supplemented with 10% fetal bovine serum and 1% penicillin-streptomycin (Gibco/Invitrogen) at 37°C in 5% CO_2_ atmosphere. Cells were maintained at about 3×10^5^ cells/ml in 100 mm culture dishes. Exponentially growing cells were used. Experiments were performed in at least triplicates.

### IM treatment

Cells were treated with IM (Biomol GmbH, Hamburg, Germany) in concentrations of 0.25 to 10 µM for 24 h (K562, LAMA-84), 48 h (HL-60, U937, U937p210BCR-ABL/c6^Tet-ON^) and 6 d (NHDF, UROtsa). Untreated cells served as controls.

### Western blot analysis, antibodies

Approximately 1×10^7^ cells per sample were incubated on ice for 10 min in 100 µl lysis buffer containing 50 mM Tris-HCl pH 7.4, 150 mM NaCl, 1 mM EDTA pH 8.0, 1% Triton X-100, 1 mM PMSF (Sigma-Aldrich, Steinheim, Germany), 2% complete protease inhibitor mix (Roche, Mannheim, Germany), 1% phosphatase inhibitor cocktails I and II (Sigma-Aldrich). Aliquots of clarified lysates were used for Bradford protein assays (ROTH, Karlsruhe, Germany). About 50–100 µg protein per lane were resolved by SDS-PAGE on BIORAD PreCast TGX 4–15% gradient gels, transferred to Immobilon-P membrane (Millipore, Bedford, USA) followed by blocking with 5% dry milk powder (AppliChem, Darmstadt, Germany) for 1 h and immunostaining with the respective primary antibody dilution for 1 to 4 h at RT or over night at 4°C. Primary antibodies (1∶1000 dilutions): anti-Separase rabbit polyclonal antibody (#PA1-46455; Pierce Biotechnology, Rockford, USA) or mouse monoclonal antibody XJ11-1B12 (#MA1-16595; Pierce Biotechnology) detecting the 220 KDa full length separase; anti-CyclinB1 monoclonal mouse antibody (#MA1-46103; Pierce Biotechnology); anti-phospho-Separase-S1126 rabbit polyclonal antibody (#AP3247a, Abgent, San Diego, USA); anti-phospho-CrkL (Tyr207) polyclonal rabbit antibody (#3181, Cell Signaling Technology, Danvers, USA); anti-ABL1 monoclonal mouse antibody (#554148, BD Biosciences, Heidelberg, Germany); anti-Securin monoclonal mouse antibody (#WH0009232M1; Sigma-Aldrich); anti-PP2A A subunit rabbit mAb (81G5) (#2041; Cell Signaling). Anti-Actin pan Ab-5 mouse monoclonal antibody (#MS-1295; Lab Vision NeoMarkers, Fremont, USA) or anti-Actin (13E5) rabbit mAb HRP Conjugate (#5125; Cell Signaling) were diluted 1∶10000. Signals were visualized with a ChemiDoc™ XRS+ System (BIO-RAD, München, Germany) after secondary antibody staining (goat anti-mouse IgG HRP conjugate antibody, goat anti-rabbit IgG HRP conjugate antibody (1∶10000, Santa Cruz, Heidelberg, Germany) utilizing SuperSignal®West Maximum Sensitivity Substrate (Thermo Fisher Scientific, Bonn, Germany). Image acquisition and densitometric analysis was performed using Image Lab™ Software (version 3.0.1, BIO-RAD). All values were normalized with Actin as loading control. Image cropping and tonal adjustments across the entire image were performed with Adobe Photoshop CS4 (Adobe Systems Inc., San Jose, CA, USA)

### Quantification of separase transcripts by qualitative reverse transcriptase PCR (qRT-PCR)

Total RNA was extracted using RNeasy kit (Qiagen, Hilden, Germany) and reverse transcribed using Superscript II kit (Gibco/Invitrogen). For quantification of separase (ESPL1) transcript levels, the commercial Hs_ESPL1_1_SG QuantiTect Primer Assay (QIAGEN) was employed according to the instructions (two-step Light Cycler 480 protocol) of the manufacturer. For normalization, the housekeeping gene glucose-6-phosphate dehydrogenase (G6PD, primer set Hs_G6PD_1_SG, Quiagen) was amplified. QRT-PCR was performed with the Roche LightCycler 480 System, using LC480 DNA Master SYBR Green and the standard LightCycler protocol (Roche Diagnostics, Mannheim, Germany). In brief, 2 µl of cDNA (an about 50 ng RNA equivalent) were added to 18 µl of reaction mix containing primers at 0.2 µM for the separase target and at 0.25 µM for G6PD in LightCycler® FastStart DNA Master^PLUS^ SYBR Green I ready-to-use hot-start PCR mix with *Taq* DNA polymerase (Roche Diagnostics) diluted with purified water according to the manufacturer's protocol. Relative transcript levels calculated from triplicate measurements were expressed as ratio separase/g6pd.

### Cell cycle analysis

Subconfluent cells were harvested and washed in 1×phosphate buffered saline (PBS), subsequently fixed in icecold 75% ethanol and stained with propidium iodide (10 µg/ml). DNA content was measured by fluorescence-activated cell sorting (FACS) using a flow cytometer FACScalibur (Becton Dickinson, San José, USA).

### Karyotype analysis

was performed as described previously [15]. At least 10 metaphases out of six cultures were analyzed by G-banding technique and interpreted according to the International System for Human Cytogenetic Nomenclature (ISCN 2009).

### Indirect immunofluorescence

Cellular distribution of Separase and centrosomal status was analyzed by immunfluorescence microscopy as described previously [15], [17]. Centrosomes were stained with rabbit anti-pericentrin polyclonal rabbit antibody (#PRB-432C, Covance, München, Germany; dilution 1∶1000). For Separase staining identical antibodies as in Western blot analysis diluted 1∶250 in blocking solution were used. After three 5 min washes in 1×PBS cells were incubated with secondary antibody Alexa Fluor 488 anti-mouse and Alexa Fluor 555 anti-rabbit (1∶500; Life Technologies, Darmstadt, Germany). For mitotic spindles, alpha-tubulin costaining was performed (#T6074, 1∶500 dilution; Sigma-Aldrich). Nuclei were stained with HOECHST33342 (#H1399, 1∶100,000; Life Technologies).

### Separase activity assay

About 60 µg cleared native protein lysate was analyzed in a quantitative fluorogenic assay according to Basu et al. [54]. Spectrofluorometry was performed in 96 well Optiplate96F plates (Greiner-Bio-One, Frickenhausen, Germany) using the Multilabel Reader Envision 2102 (PerkinElmer, Shelton, USA) at λex = 405 nm and λem = 465 nm.

### Statistical analysis

Statistical significance of unpaired data was analyzed by the Student's t-test using the GraphPad Prism software version 5.0 (GraphPad Inc., La Jolla, USA). Values of p<0.05 were considered significant.
